# Melanopsin Gene Polymorphism I394T Is Associated with Pupillary Light Responses in a Dose-Dependent Manner

**DOI:** 10.1371/journal.pone.0060310

**Published:** 2013-03-28

**Authors:** Shigekazu Higuchi, Akiko Hida, Sei-ichi Tsujimura, Kazuo Mishima, Akira Yasukouchi, Sang-il Lee, Youhei Kinjyo, Manabu Miyahira

**Affiliations:** 1 Department of Human Science, Faculty of Design, Kyushu University, Minami-ku, Fukuoka, Japan; 2 Department of Psychophysiology, National Institute of Mental Health, National Center of Neurology and Psychiatry, Kodaira, Tokyo, Japan; 3 Department of Information Science and Biomedical Engineering, Kagoshima University, Kagoshima, Kagoshima, Japan; University of Pennsylvania School of Medicine, United States of America

## Abstract

**Background:**

Melanopsin-containing intrinsically photosensitive retinal ganglion cells (ipRGCs) play an important role in non-image forming responses to light, such as circadian photoentrainment, light-induced melatonin suppression, and pupillary light response. Although it is known that there are some single nucleotide polymorphisms (SNPs) in the melanopsin (*OPN4*) gene in humans, the associations of the SNPs with non-image forming responses to light remains unclear. In the present study, we examined the associations of melanopsin gene polymorphisms with pupillary light response.

**Methods:**

Japanese university students (mean age: 21.0±1.7 years) with the genotypes of TT (n = 38), TC (n = 28) and CC (n = 7) at rs1079610 (I394T) located in the coding region participated in the present study. They were matched by age and sex ratio. Dark-adapted pupil size (<1 lx) was first measured. Then steady-state pupil size was measured during exposure to five lighting conditions (10 lx, 100 lx, 1000 lx, 3000 lx, 6000 lx in the vertical direction at eye level).

**Results:**

Significant interaction between the genotype of I394T (TT versus TC+CC) and luminance levels was found in pupil size. Under high illuminance levels (1000 lx, 3000 lx and 6000 lx), pupil sizes in subjects with the C allele were significantly smaller than those in subjects with the TT genotype. On the other hand, pupil size in subjects with the C allele under low illuminance (<1 lx) was significantly larger than that in subjects with the TT genotype. Percentages of pupil constriction under high illuminance levels were significantly greater in subjects with the C allele than in subjects with the TT genotype.

**Conclusions:**

Human melanopsin gene polymorphism I394T interacted with irradiance in association with pupil size. This is the first evidence suggesting a functional connection between melanopsin gene polymorphism and pupillary light response as an index of non-image forming response to light.

## Introduction

A new photoreceptor expressing the photopigment melanopsin was discovered in the mammalian outer retina and was named intrinsically photosensitive retinal ganglion cell (ipRGC) [1]. The ipRGCs detect irradiance of ambient light, and the signal is transmitted to the brain centers for non-image forming responses to light such as the suprachiasmatic nucleus (SCN), intergeniculate leaflet (IGL), and olivary pretectal nucleus (OPN) [2]. The ipRGCs play important roles in circadian photoentrainment [3], pupillary light response [4], [5], sleep [6], [7] and other behavioral and physiological functions. Since the discovery of this novel photoreceptor, numerous studies have been carried out to identify the functional roles of melanopsin and/or ipRGCs using transgenic mice such as mice lacking rods/cones [8] and mice lacking melanopsin [5], [9].

In humans, there are some genetic variations in the melanopsin (*OPN4*) gene according to the database of the International HapMap Project. One study showed that a single nucleotide polymorphism (SNP) of the melanopsin gene was associated with prevalence of seasonal affective disorder (SAD) [10]. It is known that a short photoperiod in winter increases the risk of SAD [11]. Although those studies suggest a functional connection between melanopsin gene polymorphism and phototransduction of a non-image forming process, there is no evidence to connect them. In addition to genetic variations, there are large inter-individual phenotypic variations in non-image forming effects of light [12]–[14]. Although it is known that dark- and light-adapted pupil sizes in normal healthy subjects have large inter-individual differences [12], [15], [16], genetic factors of inter-individual pupil size have not been elucidated.

In humans, many studies have demonstrated that pupillary light response reflects the response of ipRGCs [4], [17]–[19]. It has been reported that pupil response after light offset, which is called post illumination pupillary response (PIPR), reflect the response of iPRGCs [4], [20]. However, this method needs intense light stimuli, and pupil response measured by this method does not necessarily reflect pupil size under a real living environment of light. On the other hand, it has been reported that steady-state pupil size reflects the response of ipRGCs [18], [19], [21]. In the present study, we examined whether the *OPN4* polymorphism in a young Japanese population is associated with steady-state pupil size during exposure to light since we wanted to know pupil size in a real living environment.

## Methods

### Subjects

A total of 193 healthy Japanese university students with normal color vision volunteered to participate in this study. All participants completed the morningness-eveningness questionnaire (MEQ) [22], Pittsburgh Sleep Quality Index (PSQI) [23] and Seasonal Pattern Assessment Questionnaire (SPAQ) [11]. The Japanese versions of all questionnaires were used in the present study according to previous studies [24]–[26]. Scalp hairs were collected from each subject and used for genotyping of SNP of rs1079610 (I394T) located in the cording region. The numbers of subjects with TT, TC and CC genotypes of I394T were 129, 52 and 7, respectively. The T and C allele frequencies of I394T were 82.4% and 17.6%, respectively. Genotype frequency of I394T was consistent with the Hardy-Weinberg equilibrium (χ^2^ = 0.53, ns). Subjects gave written informed consent for participation in the study, which was approved by the Ethical Committee of Kyushu University and the Ethics Committee of the National Center of Neurology and Psychiatry.

Seventy-three subjects were recruited for measuring pupillary response to light. The three genotypes (TT, TC and CC) of I394T were carefully matched by age and sex ratio (Table 1). The numbers of subjects with TT, TC and CC genotypes were 38 (19 men, 19 women), 28 (13 men, 15 women), and 7 (3 men, 4 women), respectively. Exclusion criteria included medication or drug consumption and shift work. No significant differences in age, male/female distribution, MEQ score, PSQI score and GSS were found between subjects with the three genotypes of I394T.

**Table 1:** Demographical characteristics of subjects with three genotypes of I394T.

	TT	TC	CC	P-value	
Number (%)	38	28	7		
Age (years)	21.0±1.9	20.9±1.6	21.7±1.5	0.489	ns
Sex (M/F)	18/18	14/16	3/4	0.922	ns
MEQ score	49.3±6.7	48.2±8.6	43.3±6.7	0.165	ns
PSQI	5.57±2.38	5.57±2.20	5.00±2.58	0.828	ns
GSS	4.68±5.63	5.07±5.33	2.57±4.43	0.552	ns

Data are expressed as means ± SD.

### Genotyping

Genomic DNA was extracted from a hair using a DNA Extractor FM Kit (Wako Pure Chemical Industries, Ltd., Osaka, Japan). The *OPN4* polymorphism of rs1079610 (I394T) was genotyped in 188 subjects by the TaqMan SNP Genotyping Assay (Applied Biosystems, Foster City, CA) according to the manufacturer's procedure. Genotyping Assay ID was C__1736425-1 for rs1079610 (I394T). An attempt was made to genotype the SNP of rs2675703 (P10L) associated with seasonal affective disorder [10] by using a TaqMan probe. However, this assay did not seem to be good enough for genotyping P10L. This may be due to the fact that the SNP of rs11202106 is present in the base immediately following P10L. Genotype frequency of P10L was not consistent with the Hardy-Weinberg equilibrium (χ^2^ = 56.5, p<0.01). We therefore excluded this polymorphism from further analysis.

### Experimental conditions and procedures

After dark-adapted pupil size (20 min, <1 lx) had been measured by using an electronic pupillometer (FP-10000, TMI, Saitama, Japan), the subjects were exposed to five lighting conditions for at least five minutes (10 lx, 100 lx, 1000 lx, 3000 lx and 6000 lx). Then steady-state pupil size was measured twice for 5 sec during exposure to light. The order of lighting condition was set from the lowest illuminance (10 lx) to the highest illuminance (6000 lx). Absolute pupil size and percentage of pupil constriction based on pupil size at <1 lx were used.

**Figure 1:**
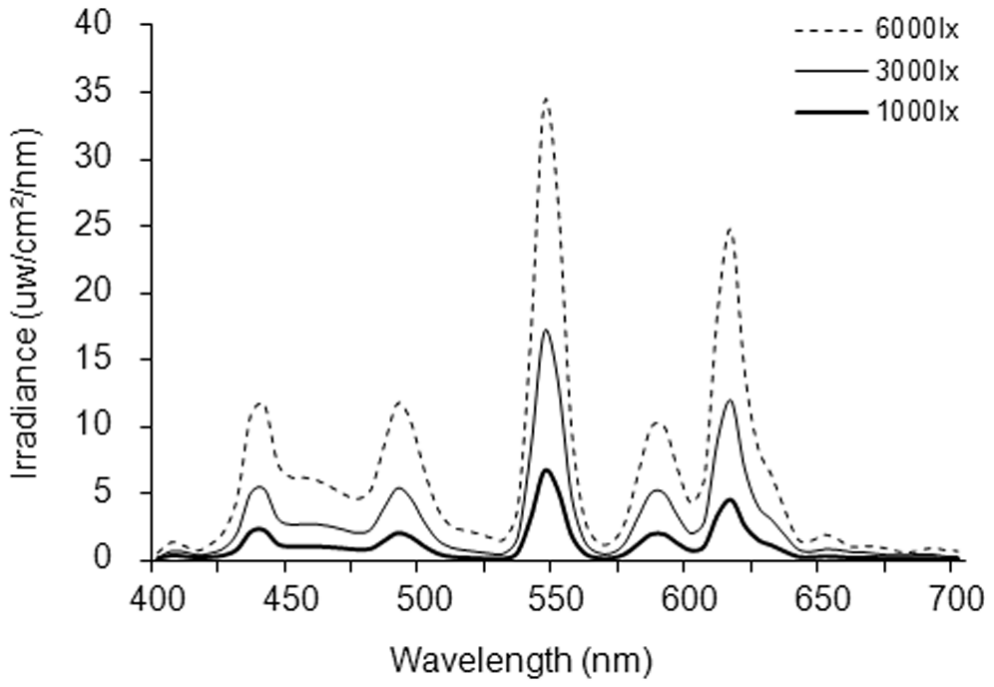
Spectral irradiance measured at eye level.

The illuminance levels were measured in the vertical direction at eye level by using an illuminance meter (CL-200, KONICA MINOLTA HOLDINGS, INC., Tokyo, Japan). White fluorescent lamps (4200 K) (FHF32EX-N-H, Panasonic Corporation, Japan) on the ceiling in the experimental room were used as light sources. Luminance levels of a white side wall at which the subjects gazed were approximately 0.5 cd/m^2^, 5.1 cd/m^2^, 42.2 cd/m^2^ 435.1 cd/m^2^, 1893.0 cd/m^2^, and 6196.0 cd/m^2^ (LS-110, KONICA MINOLTA HOLDINGS, INC., Tokyo, Japan). Spectral irradiance at the subject's eye level was also measured (LightSpex, McMahan Research Laboratories, Chapel Hill, NC) (Fig. 1). Total irradiances of 100 lx, 1000 lx, 3000 lx and 6000 lx were 27.6 µW/cm^2^, 349.8 µW/cm^2^, 897.2 µW/cm^2^ and 1892.9 µW/cm^2^, respectively. Spectral irradiances of 10 lx and <1 lx were not able to be measured because of technical difficulties.

### Statistical analysis

Two-way analysis of variance (ANOVA) followed by Bonferroni's post hoc test were performed to determine significance (p<0.05).

## Results

**Figure 2:**
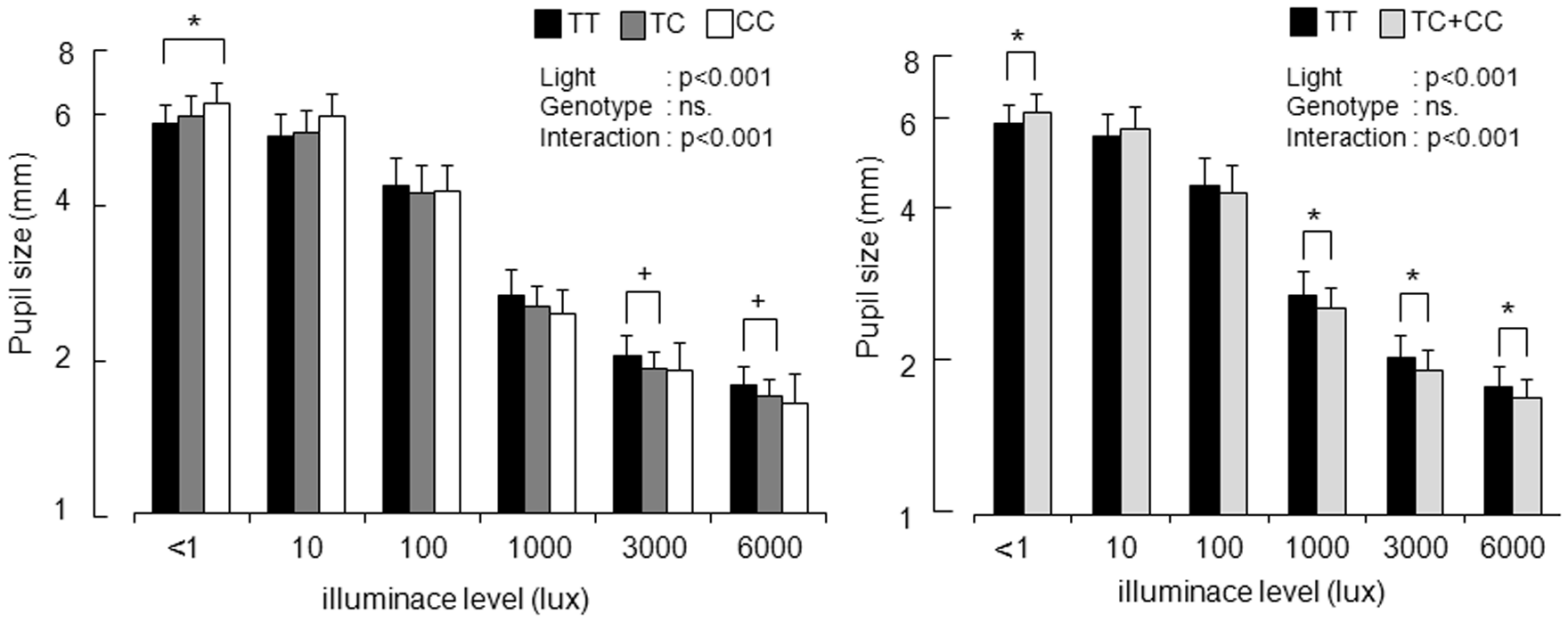
Comparison of pupil sizes (means+SD) under six illuminance levels in subjects with three (left) and two (right) genotypes of I394T. Significant interaction between illuminance and genotype was found. Pupil size in subjects with the C allele under low illuminance light (<1 lx) was significantly larger than that in subjects with the TT genotype. On the other hand, pupil sizes in subjects with the C allele under high illuminance light conditions (1000 lx, 3000 lx and 6000 lx) were significantly smaller than those in subjects with the TT genotype. *:p<0.05, +:p<0.10.

Results of two-way ANOVA (six levels of illuminance × three levels of genotype) showed that there were main effects of illuminance (F = 2746.4; df = 2.5, 176.0; p<0.0001) and a significant interaction between illuminance and genotype (F = 5.24; df = 5.0, 176.0; p<0.0001), although the main effects of genotype were not significant (F = 0.57; df = 2, 70; p = 0.566). Pupil size in subjects with the CC genotype under low illuminance light (<1 lx) was significantly larger than that in subjects with the TT genotype (p<0.05) (Fig. 2). Under high illuminance levels of 3000 lx and 6000 lx, pupil size in subjects with the TC genotype tended to be smaller than that in subjects with the TT genotype (p<0.1).

Next, the data for the TC genotype and CC genotype were combined as C-positive (Fig. 1, right). The results of two-way ANOVA (six levels of illuminance×two levels of genotype) showed that there were main effects of illuminance (F = 4233.6; df = 2.5, 176.8; p<0.0001) and a significant interaction between illuminance and genotype (F = 8.03; df = 2.5, 176.8; p<0.0001). Pupil size in subjects with the C allele under low illuminance light (<1 lx) was significantly larger than that in subjects with the TT genotype (p = 0.040). On the other hand, pupil sizes in subjects with the C allele under high illuminance light at 1000 lx, 3000 lx and 6000 lx were significantly smaller than those in subjects with the TT genotype (p = 0.031, p = 0.011 and p = 0.013, respectively).

**Figure 3:**
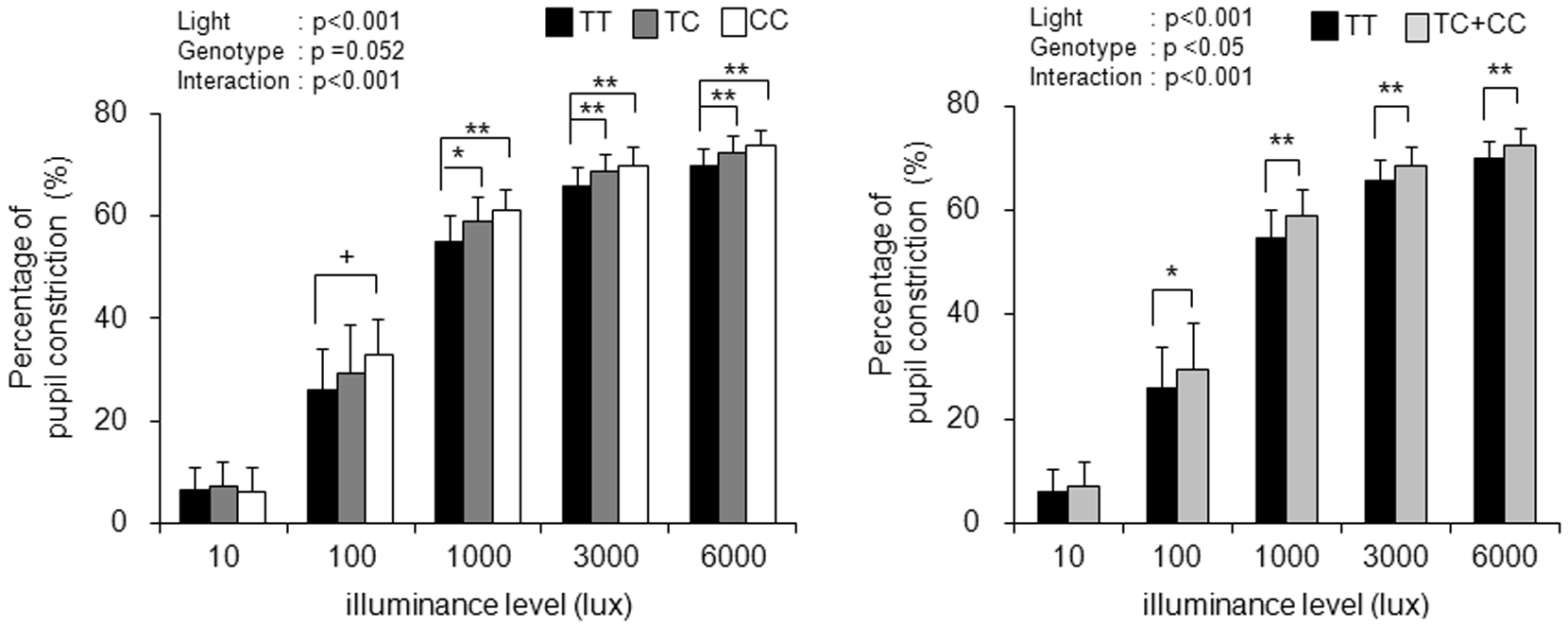
Percentages of pupil constriction (mean+SD) in subjects with three genotypes (left) and two genotypes (right) of I394T. Main effects of genotype and illuminance level were found. The percentages of pupil constriction in subjects with the C allele of I394T were significantly greater than those in subjects with the TT genotype under high illuminance level. **:p<0.01, *:p<0.05.

Based on the pupil size at <1 lx, the percentages of pupil constriction were calculated (Fig. 3). Results of two-way ANOVA (five levels of illuminance×three levels of genotype) showed that there were main effects of illuminance (F = 2312.4; df = 2.0, 137.6; p<0.0001) and genotype (F = 7.29; df = 2, 70; p<0.001). The interaction between illuminance and genotype tended to be significant (F = 2.43; df = 3.9, 137.6; p = 0.52). The percentages of pupil constriction in subjects with the CC and TC genotypes were significantly higher than those in subjects with the TT genotype. No significant differences were found between percentages of pupil constriction in subjects with the CC genotype and subjects with the CT genotype. As for the combined data (TC+CC), there were main effects of illuminance (F = 3708.1; df = 2.0, 142.0; p<0.0001) and genotype (F = 12.84; df = 1, 71; p<0.001) and a significant interaction between them (F = 3.12; df = 2.0, 142.0; p = 0.47). The percentages of pupil constriction in subjects with the C allele were significantly higher than those in subjects with the TT genotype under inlluminance level of 100 lx or higher (Fig. 3, right).

## Discussion

It was found that melanopsin gene polymorphism I394T interacted with irradiance in association with pupil size. Pupil sizes in subjects with the C allele (TC genotype+CT genotype) under high illuminance conditions (3000 lx and 6000 lx) were significantly smaller than those in subjects with the TT genotype (Fig. 1). The percentage of pupil constriction in subjects with the C allele was significantly larger than that in subjects with the TT genotype (Fig. 2). In the present study, the effects of genotype at high light intensity are consistent with the feature of melanopsin response to light since ipRGCs respond to high-intensity light [3]. Furthermore, pupillary light reflex diminishes at high irradiances in melanopsin-knockout mice [5]. In the present study, the small pupil size and large constriction in subjects with the C allele suggest a functional connection between human melanopsin gene polymorphism and pupillary light response.

In contrast to the results under a high illuminance level, pupil size in subjects with the C allele under low illuminance (<1 lx) was significantly larger than that in subjects with the TT genotype. Since ipRGCs respond to high-intensity light [3], the association found in a very low illuminance condition is inconsistent with the feature of melanopsin. Recently, it has been reported that signals from rods were transmitted to ipRGCs and that these signals caused pupillary light response [27]. Furthermore, an important role of rods under very dim light conditions for non-image forming effects of light has been reported [28]. However, contribution of melanopsin to signal transduction of ipRGCs from rods is unknown and further study is needed to confirm this relationship.

An association with pupillary light response was found for the SNP of rs1079610 (I394T) in the present study. This region is different from that in a previous study showing a significant association between SNP of the melanopsin gene and prevalence of SAD [10]. In the previous study, prevalence of SAD was associated with SNP of rs2675703 (P10L) but not with SNP of rs1079610 (I394T). Recently, It has been reported that SNP of P10L is associated with sleep onset time as a function of daylength [29]. The association with genotype of P10L and pupillary light response should be tested in a future study since genotyping SNPs of P10L was insufficient due to technical limitations in the present study.

Interestingly, there are geographic and/or ethnic differences in allele frequency of I394T. According to the database of International HapMap Project, C allele frequency of I394T in the European population (34.2%) is larger than that in the Asian-Japanese population (17.0%) and that in the Sub-saharan African population (14.2%). Why is C allele frequency larger in the European population? It has been reported that levels of intraocular straylight (IOSL) are higher in subjects with light-blue colored irises in European people [30] and that the prevalence rate of age-related macular degeneration is higher in European people [31]. Furthermore, there is some evidence suggesting ethnic differences in SAD [32] and light-induced melatonin suppression [33]. In the present study, subjects with the C allele had small pupils and large constriction under a bright light condition. In European people, there might be some advantages of having these properties. In order to examine this speculation, studies using other ethnic groups should be carried out to determine whether the genotype-phenotype association persists.

In the present study, we used steady-state pupil response to white fluorescent light. Although even steady-state pupil responses reflect the response of ipRGCs [18], [21], the measurement of PIPR is expected in future studies since measurement of PIPR has been reported to be useful for identifying the ipRGC response in humans with normal vision [4], [34], [35]. Furthermore, since ipRGCs are sensitive to light of a short wavelength (blue light) [3], [36], using monochromatic light of different wavelengths would clarify the difference in pupillary light response between genotypes of *OPN4*.

Finally, ipRGCs are thought to contribute to non-image forming effects of light such as circadian photoentrainment and light-induced melatonin suppression in humans [37], [38]. Recently, a new method using a silent-substitution technique was used to stimulate ipRGCs independently of rods and cones [18], [39]–[41]. Therefore, further studies are needed to examine the association between *OPN4* polymorphism and human physiological responses to light in various ways in order to clarify the functional role of ipRGCs in humans.

## Conclusions

Human melanopsin gene polymorphism I394T interacted with irradiance in association with pupil size. This is the first evidence suggesting a functional connection between melanopsin gene polymorphism in humans and pupillary light response as an index of non-image forming response to light.
